# Colon Mass as a Secondary Metastasis from Cholangiocarcinoma: A Diagnostic and Therapeutic Dilemma

**DOI:** 10.7759/cureus.707

**Published:** 2016-07-22

**Authors:** Azfar Niazi, Muhammad W Saif

**Affiliations:** 1 Outcomes Research, Cleveland Clinic Foundation; 2 Hematology/Oncology, Tufts Medical Center, Tufts University School of Medicine

**Keywords:** colon cancer, cholangiocarcinoma, metastases

## Abstract

Cholangiocarcinoma (bile ducts cancer) is a rare and aggressive form of cancer. It metastasizes frequently to liver, peritoneum, and lungs. Colon metastasis is extremely uncommon. We report here a 70-year-old male who was diagnosed with cholangiocarcinoma for which he underwent a Whipple procedure. Fifteen months later, a CT scan revealed mural thickening in the colon; this was supplemented with a PET scan, which confirmed this mass. Histological diagnosis of metastatic cholangiocarcinoma to the colon was made and the patient was treated with chemotherapy. Although rare, cholangiocarcinoma metastasis can be found in the colon. A high index of suspicion is required to diagnose and treat early. More cases need to be reported to find out further about the prognosis of the disease.

## Introduction

Cholangiocarcinoma, also known as bile duct cancer, originates from bile ducts, including the intrahepatic, perihilar, or distal (extrahepatic) biliary tree, exclusive of the gall bladder or ampulla of Vater. Cholangiocarcinoma accounts for about 3% of gastrointestinal malignancies. It is a rare form of cancer that usually is locally advanced at the time of diagnosis. Intrahepatic cholangiocarcinoma arises from small intrahepatic ductules (peripheral cholangiocarcinomas) or large intrahepatic ducts proximal to the bifurcation of the right and left hepatic ducts. The extrahepatic bile ducts are divided into perihilar (including the confluence itself) and distal segments, with the transition occurring at the point where the common bile duct lies posterior to the duodenum [1]. The reported incidence in the US is one to two cases per 100,000 of the population. Approximately 2,600 cases of intrahepatic and 2,000 to 3,000 cases of extrahepatic cholangiocarcinoma are diagnosed annually in the US [2]. Generally, the incidence of biliary tract cancers increases with age; the typical patient with cholangiocarcinoma is 50 to 70 years old. It is more common in males. In the US and Europe, the main risk factors are primary sclerosing cholangitis and choledochal cysts. More than 90% of these are adenocarcinomas and the rest are mostly squamous cell carcinomas. They are also graded as moderately or poorly differentiated. Adenocarcinomas are further divided into three types: nodular, sclerosing, and papillary. There is no pathognomic immunohistochemical test that can be used to confirm the cell type of origin. However, positive results of several types of immunohistochemical staining (cytokeratin, carcinoembryonic antigen, and mucin) may be used to support a diagnosis of malignant biliary epithelium. In particular, cytokeratin-7 (CK-7) positivity is consistent with biliary tract origin.

The most common sites of metastases are liver, peritoneum, and lungs but cases in unusual sites, such as skin and adrenal gland, have been reported previously [3-5]. Colon metastasis of cholangiocarcinoma is very rare, and only three cases have been reported thus far with metastases [6-8] and one case with infiltration [9]. We report the fourth case of metastasis from cholangiocarcinoma to the colon to bring awareness about the proper diagnosis and management of uncommon sites of metastases as it poses a diagnostic as well as a therapeutic challenge to both patients and the treating physicians.

Informed patient consent was obtained prior to treatment.

## Case presentation

**Figure 1:**
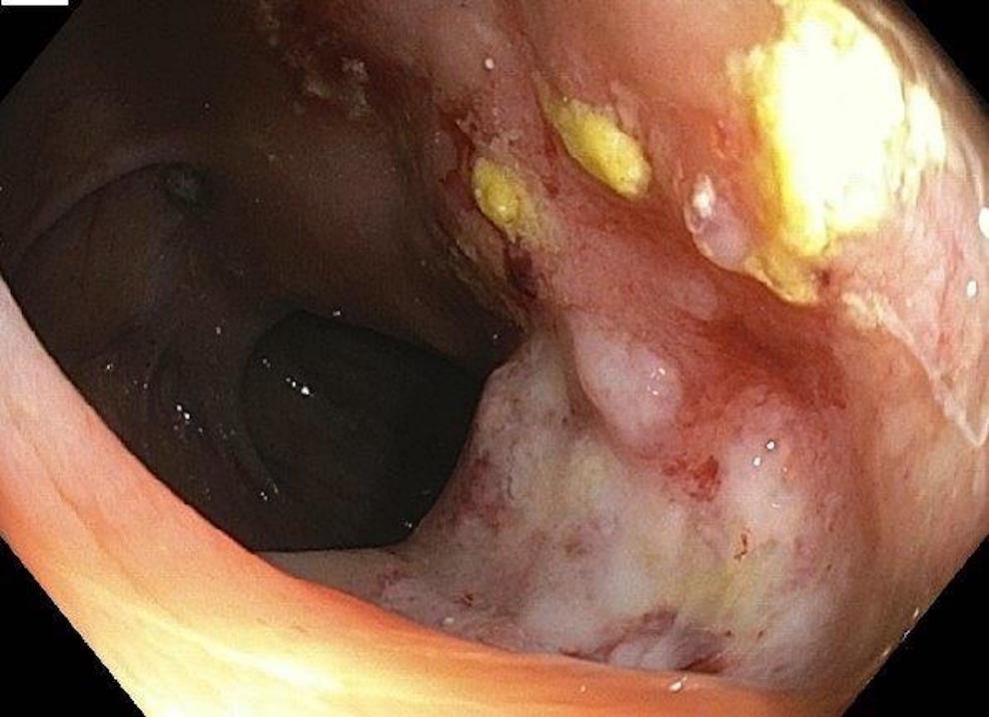
Colonoscopic Findings Colonoscopy showing a mass in the ascending colon as previously noticed on the CT scan and confirmed to be a metastasis from primary cholangiocarcinoma.

We diagnosed a 70-year-old-male with cholangiocarcinoma for which he underwent a Whipple procedure. The pathology report showed a moderately differentiated adenocarcinoma of the middle and distal bile duct, 0.5 cm in size, which was confined to the bile duct with clear margins; one out of 17 (1/17) lymph nodes tested positive for adenocarcinoma. With that information, we staged the tumor as pT1N1MX (Stage IIB). Subsequently, the patient was placed on adjuvant chemotherapy consisting of gemcitabine, 1,000 mg/m^2^ weekly for three out of four weeks each month for a total of six months. Nine months after the therapy started, the patient developed atrial fibrillation for which he underwent cardioversion and was then put on Coumadin. After being on Coumadin for five months, he noticed blood in his stool. An investigation revealed an elevated INR of 5.2. After correcting the INR, he underwent a CT scan as well as a colonoscopy due to continued bleeding. The CT scan of the abdomen and pelvis showed an area of mural thickening in the middle to distal portion of the ascending colon suspicious for a neoplastic process, bilateral pleural effusion more than the previous studies performed earlier. Colonoscopy confirmed the mass initially noticed on the CT scan as shown in Figure 1. 

Histology showed adenocarcinoma, and the immunostaining was positive for CK7 and CA19-9 and negative for CEA and CK20. These pathological findings were consistent with a metastatic bile duct adenocarcinoma confirmed by an immunoperoxidase staining pattern identical to the patient’s primary tumor at the time of initial diagnosis about 15 months previously. A PET-CT scan revealed a small focus of tracer uptake within the right supraclavicular area with a standardized uptake value (SUV) of 2.5. A pretracheal lymph node had an SUV of 3.3, a subcarinal lymph node had an SUV of 4.0, and a right hilar lymph node had an SUV of 3.1. Mild tracer uptake was seen in the left lower effusion, but no uptake in the right pleural effusion. In the right lower lobe, a focal mass-like lesion was found with SUV of 2.4. The tracer uptake was insignificant in the liver, but there was significant tracer uptake seen in the bowel adjacent to the gastric anastomosis with an SUV of 3.7, which was possibly due to inflammatory change. The lymph node anterior to the inferior vena cava within the right mid-abdomen had an SUV of 2.7. A soft tissue mass abutting the colon at the level of the hepatic flexure had an SUV of 11.1. A lymph node anterior to the right common iliac vein had an SUV of 3.6. The major conclusion of the PET-CT was that the soft tissue mass near the hepatic flexure was consistent with malignancy. The adenopathy in the abdomen, the chest findings, and the lesion in the right lower lobe of the lung all pointed toward metastasis.

The case was thoroughly discussed in different multimodality conferences and finally diagnosed as colon metastasis from his primary cholangiocarcinoma. The patient was treated with systemic chemotherapy consisting gemcitabine and cisplatin based on the diagnosis of cholangiocarcinoma, which resulted in a partial response with a duration of response of four months. He later progressed to peritoneal disease and was treated with oral capecitabine.

## Discussion

Only a handful cases have been published to date to verify the spread of cholangiocarcinoma to unusual locations, such as adrenal glands, bones, brain, colon, and skin [3-5]. One patient, who was diagnosed with cholangiocarcinoma based upon the histopathology of an intrahepatic mass, was treated with GEMOX (gemcitabine and oxaliplatin) followed by 5-fluorouracil and mitomycin-C; however, nothing kept the tumor from spreading to the brain. After the metastasis to the brain, he received 300 cGy radiation to the whole brain but the family refused the treatment after one dose of the treatment. The patient died 17 days after the last treatment [6]. Another patient presented with an intestinal obstruction caused by metastasis that manifested six years after surgery for intrahepatic cholangiocarcinoma (ICC), which was confirmed by histopathology. There had been no signs of recurrence until an increase in the CA19-9 level was detected six years later. Colonoscopy revealed an ulcer-like lesion and stenosis at the level of the hepatic flexure. The patient subsequently presented with abdominal pain and underwent a right hemicolectomy with partial resection of the hepatic segment V. The immunohistological finding of the expression pattern of cytokeratins and mucins was consistent with the pathology of cholangiocarcinoma at the time of initial diagnosis rather than the diagnosis of a colon cancer origin [7]. Similarly, a case of mass-forming type intrahepatic ICC with direct infiltration of the transverse colon and sequential brain metastasis. The patient was treated by a curative right hepatectomy with right hemicolectomy, followed by resection of the brain metastasis; there has been no evidence of recurrence in the seven years since the hepatic resection. This case suggested that, in selected patients, surgical resection may improve the prognosis of ICC involving the adjacent organs, even with brain metastasis [8]. Our patient presented with mural thickening of the colon on CT scan 15 months after the Whipple procedure. PET scan showed multiple metastases. Diagnosis of metastatic cholangiocarcinoma was made based on tissue obtained via colonoscopy, which showed thickening of the hepatic flexure but no clear mass. The pathology report was consistent with his initial diagnosis of cholangiocarcinoma.

Another important aspect of this case is that our patient developed atrial fibrillation for which he was placed on Coumadin. Bleeding from the rectum or other sites of bleeding is not an unusual finding in patients receiving anticoagulation therapy, including Coumadin. However, due to our expertise in GI malignancies and experience of reporting similar cases, we did not stop after correcting INR but further investigated the cause of bleeding that led to this diagnosis [10]. This is an important lesson as we have more chemotherapeutic agents available to these patients and growing data on newer agents, such as nab-paclitaxel, in this setting.

## Conclusions

In summary, cholangiocarcinoma is a rare but aggressive form of cancer that spreads rapidly unless it is completely resected. It is usually diagnosed at a stage when it is too advanced for surgical resection. The tumor is relatively silent in the start and usually diagnosed when it is in an advanced stage. There is, however, the need for a screening method to detect the tumor at its early stages. Some patients do present with jaundice, and primary care providers refer them for ultrasound and endoscopic retrograde cholangiopancreatography. But the patients presenting with vague abdominal pain delay the initial diagnosis of such patients. Therefore, a high index of suspicion is required for early detection. It is usually late and the tumor is advanced by the time jaundice develops. Last but the least, rapid referral and awareness of unusual sites of metastases of cholangiocarcinoma is the key in the quick diagnosis and the treatment of cholangiocarcinoma.
